# 

**Published:** 1914-03

**Authors:** 


### THE BRISTOL
### Medico-Chirurgical Journal.

A JOURNAL OF THE MEDICAL SCIENCES FOR THE WEST OF ENGLAND AND SOUTH WALES

*PUBLISHED UNDER THE AUSPICES OF*

*THE BRISTOL MEDICO-CHIRURGICAL SOCIETY.*

EDITOR:
P. WATSON-WILLIAMS, M.D.

WITH WHOM ARE ASSOCIATED
J. MICHELL CLARKE, M.A., M.D., LL.D., J. LACY FIRTH, M.S., M.D.,
AND JAMES SWAIN, M.S., M.D.

ASSISTANT-EDITOR:
J. A. NIXON, B.A., M.B.

EDITORIAL SECRETARY:
J. M. FORTESCUE-BRICKDALE, M.A., M.D.

"Scire est nescire, nisi id me

Scire alius sciret."

SURGEON GENERAL'S 24222 // LIBRARY

*VOL. XXXII.*

BRISTOL: J. W. ARROWSMITH LTD.
LONDON: J. & A. CHURCHILL, 7 GREAT MARLBOROUGH STREET.
1914.

J. M. FORTESCUE-BRICKDALE.

---

### The Library of the Bristol Medico-Chirurgical Society.

*The following donation has been received since the publication of the list in December.*

*February 28th,* 1914.

Walter C. Swayne, M.D. .. .. .. .. 1 volume.

### NINETY-FIRST LIST OF BOOKS.

The titles of books mentioned in previous lists are not repeated.

The figures in brackets refer to the figures after the names of the donors, and show by whom the volumes were presented. The books to which no such figures are attached have either been bought from the Library Fund, or received through the *Journal.*

| | | |
|---|---|---|
| **Abrahams, G. Herschell and A.** *Chronic Colitis* .. .. .. .. | | 1914 |
| *Alimentary Toxæmia, Discussion on* .. .. .. .. .. .. .. .. .. | | 1913 |
| **Basu, B. D.** .. .. | *The Dietetic Treatment of Diabetes*.. 4th Ed. | 1913 |
| **Bennett, R. A.** .. | *Rules for use of Tuberculin* .. .. .. .. .. | 1914 |
| **Cammidge, P. J.** .. | *The Fæces of Children and Adults* .. .. .. | 1914 |
| **Cole, R. H.** .. .. | *Mental Diseases* .. .. .. .. .. .. .. .. | 1913 |

**Cuff, I. Stewart and H. E.** *Practical Nursing* .. .. .. 4th Ed. 1913
**Cunningham [D. J.]** *Manual of Practical Anatomy* (Edited by A. Robinson). Vol. I .. .. .. 6th Ed. 1914
**Elmslie, R. C.** .. *Coxa Vara, its Pathology and Treatment* .. 1913
**Finzi, N. S.** .. .. *Radium Therapeutics* .. .. .. .. .. 1913
**Forbes, T. D. Luke and N. H.** *Natural Therapy* .. .. [2nd Ed.] 1913
**Gade, H. G.** .. .. *Diagnosen av Barnetuberkulosens kliniske Initialformer* .. .. .. .. .. .. .. .. 1914
**Gibson, A. G.** .. *Post-Mortem Handbook* .. .. .. .. .. 1914
**Green, C. E.** .. .. *The Cancer Problem* .. .. .. .. 3rd Ed. 1914
**Groves, E. W. H.** .. *Synopsis of Surgery* .. .. .. .. 4th Ed. 1914
**Gruner, O. C.** .. *The Biology of the Blood-Cells* .. .. .. 1913
**Hawkins-Dempster, H.** *Explanatory Lectures for Nurses* .. .. 1913
**Herschell and A. Abrahams, G.** *Chronic Colitis* .. .. .. .. 1914
**Knott, C. G.** .. .. *Physics* .. .. .. .. .. .. 3rd Ed. 1913
**Landolt, E. and M.** *Defective Ocular Movements* (Trans. by A. Roemmele and E. W. Brewerton) .. .. 1913
**Luke and N. H. Forbes, T. D.** *Natural Therapy* .. [2nd Ed.] 1913
**Macdonald, D. M.** *The Practitioner's Practical Prescriber* .. .. 1913
**Paterson, A. M.** .. *The Anatomist's Note Book* .. .. .. .. 1914
**Prichard, A. H.** .. *Practical Prescribing* .. .. .. .. .. .. 1913
**Riviere, C.** .. .. *Early Diagnosis of Tubercle* .. .. .. .. 1914
**Scott, T. B.** .. .. *The Road to a Healthy Old Age* .. .. .. 1914
**Stewart and H. E. Cuff, I.** *Practical Nursing* .. .. .. 4th Ed. 1913
**Taylor, A.** .. .. *The Sanitory Inspector's Handbook* .. 5th Ed. 1914
**Tredgold, A. F.** .. *Mental Deficiency* .. .. .. .. 2nd Ed. 1914
**Walker, J. W. T.** .. *Genito-Urinary Surgery* .. .. .. .. .. 1914
**Wallace, J. S.** .. *Dental Disease and Public Health* .. .. .. 1914
**Walters, F. R.** .. *Sanatoria for the Tuberculous* .. .. 4th Ed. 1913

### TRANSACTIONS, REPORTS, JOURNALS, &c.

American Laryngological Association, Transactions of the .. .. 1913
American Laryngological, Rhinological, and Otological Society, Transactions of the .. .. .. .. .. .. .. .. .. .. 1913
American Surgical Association, Transactions of the .. Vol. XXXI 1913
American Urological Association, Transactions of the Vol. VII 1913
Archiv für klinische Chirurgie .. .. .. .. .. .. Bds. C, CI 1913
Boston Medical and Surgical Journal, The .. Vol. CLXVIII 1913
British Journal of Children's Diseases .. .. .. .. Vol. X 1913
British Medical Journal, The .. .. .. .. .. .. .. Vol. II for 1913
Deutsche Zeitschrift für Chirurgie .. .. Bds. CXXI–CXXIV 1913
Glasgow Medical Journal, The .. .. .. .. .. .. Vol. LXXX 1913
Hospitals—
Bellevue and Allied Hospitals, New York, Medical and Surgical Reports .. .. .. .. .. .. .. .. .. .. Vol. V 1911–12
Guy's Hospital Reports .. .. .. .. .. .. .. Vol. LXVII 1913
Westminster Hospital Reports .. .. .. .. Vol. XVIII 1913

| | | |
|---|---|---|
| International Abstract of Surgery | Vol. XVI | 1913 |
| Johns Hopkins Hospital Bulletin, The | Vol. XXIV | 1913 |
| Journal of Laryngology, The | Vol. XXVIII | 1913 |
| Journal of the American Medical Association, The | Vol. LX | 1913 |
| Medical Directory, The | | 1914 |
| Medical Press and Circular, The | Vol. CXLVII | 1913 |
| Medical Record | Vol. LXXXIII | 1913 |
| Medical Who 's Who, The | | 1914 |
| Münchener medizinische Wochenschrift | Bd. II for | 1913 |
| Practitioner, The | Vol. XCI | 1913 |
| Progrès médical, Le | | 1913 |
| Progressive Medicine | Vol. IV | 1913 |
| Public Health | Vol. XXVI | 1912–13 |
| Quarterly Journal of Medicine, The | Vol. VI | 1912–13 |
| Revue de Chirurgie | Tom. XLVII | 1913 |
| Revue hebdomadaire de Laryngologie | Tom. XXXIV, Vol. II | 1913 |
| Smithsonian Report for 1912 | | 1913 |
| Studies from the Department of Pathology, College of Physicians and Surgeons, New York | Vol. XIII | 1913 |
| Surgery, Gynecology, and Obstetrics | Vol. XVI | 1913 |
| Zentralblatt für Chirurgie | | 1913 |
| Zentralblatt für Gynäkologie | | 1913 |
| Zentralblatt für innere Medizin | | 1913 |

### MEETINGS OF SOCIETIES.

### Bristol Medico-Chirurgical Society.

### Local Medical Notes.

**University of Bristol.**—Students of the University have recently passed the following examinations :—

UNIVERSITY OF LONDON.—*First Examination for Medical Degrees, Part I :* A. Douthwaite, N. Kemm.

UNIVERSITY OF BRISTOL.—*M.B., First Examination, Part I :* E. J. Ball, D. G. Cossham, Eva Richmond, Evelyn B. Salter. *Final M.B., Ch.B. :* Maurice C. Barber.

D.P.H. LOND.—W. W. Pratt.

L.D.S. ENG.—*Chemistry :* A. J. Constance, A. L. Morgan, *Physics :* S. W. A. Davis.

M.R.C.P. LOND.—Cecil Clarke, M.D., Lond., J. M. Fortescue-Brickdale, M.D. Oxon.

### APPOINTMENTS.

C. E. K. Herapath, M.D., has been appointed Medical Registrar to Bristol Royal Infirmary.

J. P. I. Harty, B.A., M.B., B.Ch., F.R.C.S., has been appointed Clinical Assistant to the Ear, Nose and Throat Department at the Bristol Royal Infirmary.

W. W. Salisbury, M.B., B.S. Lond., has been appointed Resident Medical Officer at the Hospital for Women, Soho Square, W.

F. H. Pickin, M.R.C.S., L.R.C.P., has been appointed Clinical Assistant at the St. Paul's Hospital for Skin and Urinary Disease, London.

C. A. Joll, M.B., B.S. Lond., F.R.C.S., has been appointed Honorary Assistant Surgeon at Royal Free Hospital, London.

